# Egg size and fecundity of biannually spawning corals at Scott Reef

**DOI:** 10.1038/s41598-020-68289-4

**Published:** 2020-07-23

**Authors:** Taryn Foster, James Gilmour

**Affiliations:** Australian Institute of Marine Science, Indian Ocean Marine Research Centre, University of Western Australia, Perth, WA Australia

**Keywords:** Ecology, Marine biology

## Abstract

Egg size and fecundity are often used as proxies for coral reproductive success and health. The amount of energy a coral invests in reproduction reflects its environmental conditions during gametogenesis. Additionally, assuming resources for reproduction are limited, it is thought that an increase in egg size should result in a decrease in the number of eggs produced i.e. investing in many small eggs or fewer larger eggs. The biannually spawning populations of Scott Reef offer a unique opportunity to compare the egg size and polyp fecundity of corals exposed to different environmental conditions during gametogenesis, prior to spawning in autumn (March) and spring (October). In this study, we investigated the relationship between egg size and polyp fecundity within and between seven *Acropora* species from 2008 to 2010. We also quantified the fecundity and egg size of four *Acropora* species that spawn during both autumn and spring (2008–2010). We found no seasonal variability in egg size and fecundity in the species studied here, possibly as a result of a summer light regime being impacted by high cloud cover in cyclone season. There was high natural variability and no apparent trade-off between egg size and fecundity, both within and between each species. These findings challenge the assumption that egg size and fecundity are negatively correlated, or that a simple, energetically constrained trade-off exists between the two.

## Introduction

*Acropora* corals are hermaphroditic broadcast spawners, releasing their gametes into the water column simultaneously for external fertilisation^1^. Multi-specific synchronous coral spawning has been observed since the 1980s on the Great Barrier Reef (GBR)^2^, but coral reproduction on Western Australian (WA) reefs has only been studied in detail in more recent years, and spawning patterns vary latitudinally^3–8^ . Broadcast spawning on WA reefs occurs in autumn (usually March/April) and at some reefs also in spring (October/November), but participation in spring spawning decreases with increasing latitude^3,4,6,8^. At the tropical Kimberley Oceanic reefs (Ashmore Reef, Scott Reef, and the Rowley Shoals) there are significant multi-specific spawning events in both autumn and spring, but the majority of species participate in autumn spawning^5,8,9^. For *Acropora* corals, some species or colonies spawn only in autumn, some spawn only in spring, while others participate in both spawning seasons^10^. For the biannual spawners, a similar proportion of colonies spawn in each season, but most colonies (98%) spawn only once per year and always during the same season^10^. Thus conspecifics that spawn biannually at Scott Reef are temporally isolated and rarely interbreed.

Although there is known variation in the participation of conspecific colonies in the different spawning seasons at Scott Reef^5,8,10^, it is unknown whether reproductive output differs between autumn and spring spawning populations. The environmental conditions that colonies experience through several months of gametogenesis in winter and spring (leading up to spring spawning), differ markedly from those in summer and autumn (leading up to autumn spawning), and consequently the reproductive output may vary. For example, seasonal variations in coral lipid levels (energy reserves) have been linked to seasonal difference in both light and temperature^11^. Warmer temperatures in summer elevate metabolic rate and may increase the energy available for processes such as reproduction and calcification^11,12^. At Scott Reef, mean monthly water temperatures leading up to autumn spawning (November to March) are the warmest for the year (29–30 °C) while leading up to spring spawning (June to October) they are the coolest (26–28 °C). Alternatively, high temperatures can also stress corals and reduce fecundity^13^. In the worst instances, high temperatures cause coral bleaching and the loss of zooxanthellae, reducing photosynthesis and the ability to build energy reserves. Bleaching has reduced pre-spawning lipids by 60% and significantly reduced the number of eggs produced compared to non-bleaching years^14,15^. Following the 1998 bleaching on the GBR, some coral species had no eggs, while others had eggs that were reduced in both size and number^14^. In addition to reducing energy available for reproduction, environmental variation can also cause the available energy to be re-directed to other life history traits. For example, in low light conditions energy was channeled to calcification but not reproduction^16^.

Increasing the size and number of eggs requires the use of energy that has been channeled to oogenesis (egg production). Larger eggs are fertilized at a greater rate, probably because they provide a larger target for sperm^17^. Additionally, larger eggs have more stored lipids^15^, which provide important energy reserves for the azooxanthellate larvae produced by *Acropora* corals. Until azooxanthellate larvae and new recruits are able to acquire zooxanthellae, they rely on the lipid stores provided by the parent to meet their energetic needs^18^. Consequently, these energy stores affect both larval duration (which affects geographic distribution) and post-settlement survival^18,19^. Conversely, producing more eggs has obvious life history advantages, because it increases the numerical odds of self-proliferation^15^. This suggests that there is a trade-off between producing fewer large eggs, each with an increased chance of survival and greater potential for dispersal, or many small eggs with reduced survival and dispersal^19,20^. An inverse relationship between egg size and fecundity has been reported in several studies^21–23^, however, these studies only compare between species and not within a species. The trade-off or inverse relationship between fecundity and egg size can also change in response to stress. For example, *Acropora millepora* colonies that were exposed to temperature stress, produced fewer eggs but maintained egg sizes^15^. Similarly, while both fecundity and egg size were reduced in bleached soft corals, energy allocated to the number of eggs appeared to be re-directed to maintaining fewer eggs to reach mature sizes^24^. In this study, we investigated whether there were seasonal differences in egg size and polyp fecundity in biannually spawning *Acropora* corals from Scott Reef, and whether there was an inverse or trade-off relationship between egg size and fecundity both within and between species.

## Results

### Environmental conditions

**Figure 1:**
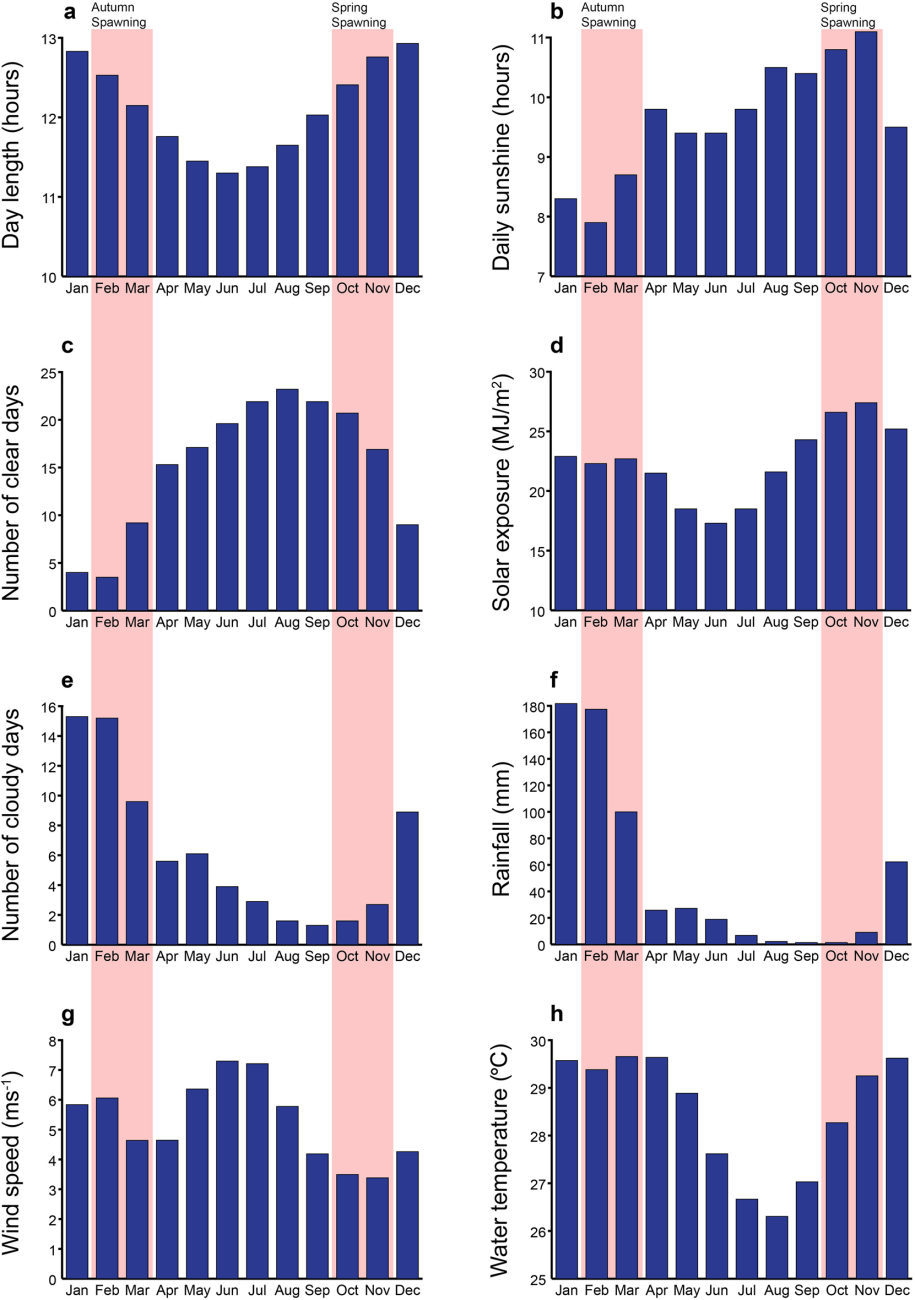
Mean monthly environmental conditions for Scott Reef (**a**,**g**–**h**) or Broome weather station (**b**–**f**). The mean monthly day lengths for Scott Reef were from the US Naval Observatory in 2016 (**a**). Wind speed data for Scott Reef was obtained from the NOAA Earth Systems Laboratory (1968–1996) (**g**) and water temperatures were from the Hadley Centre Sea Surface Temperature dataset (1990–2000) (**h**). The Australian Bureau of Meteorology Broome weather station datasets were collected during the following time periods; daily sunshine hours (**b**) from 1993 to 2016, number of clear days (**c**) from 1939 to 2010, daily solar exposure (**d**) from 1990 to 2017, number of cloudy days (**e**) from 1939 to 2010 and rainfall (**f**) from 1939 to 2017. Months leading up to autumn spawning are November to March and months leading up to spring spawning are June to October. Pink bars indicate spawning months in autumn and spring.

Scott Reef is a relatively low latitude reef (14°S), but has some seasonal variation in environmental conditions. In the months leading up to autumn spawning (November to March), the days are longer (12–13 h) and water temperatures are warmer (29–30 °C); in the months leading up to spring spawning (June to October), the days are shorter (11–12 h) and water temperatures are cooler (26–28 °C) (Fig. 1a,h). Cyclone season is from December to April, and the majority of the rainfall occurs during the summer months of January and February, with correspondingly high cloud cover during these months (Fig. 1e,f). Consequently, the overall daily solar exposure is highest in October and November rather that the months with most daylight hours (December and January), and there is relatively little annual variation in overall solar exposure (ranges from ~ 17 to 27 MJ/m^2^) (Fig. 1d).

### Egg size versus fecundity

**Figure 2:**
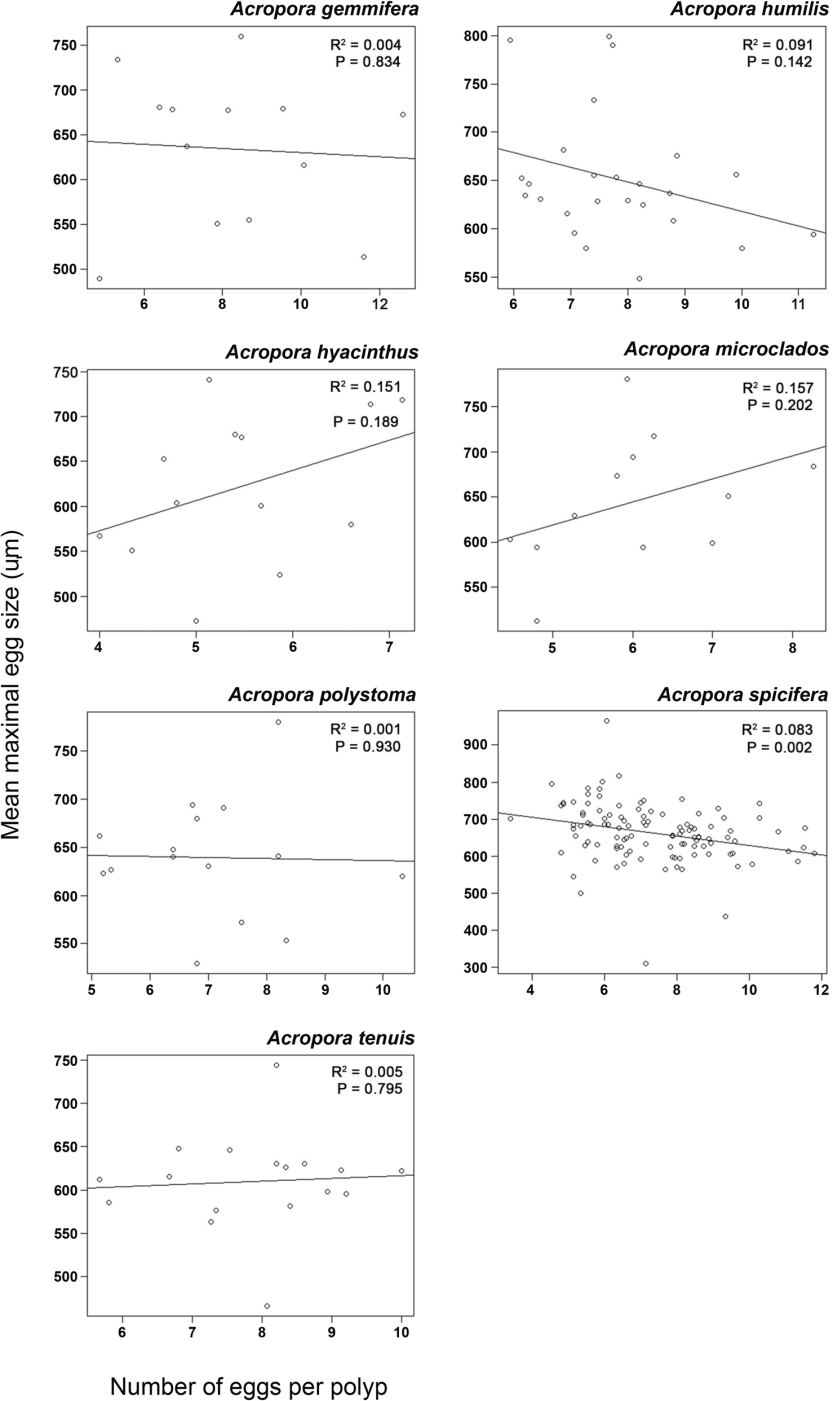
Linear regression plots for the relationship between mean maximal egg size and the number of eggs per polyp for the corals *Acropora gemmifera*, *A humilis*, *A hyacinthus*, *A microclados*, *A polystoma*, *A spicifera* and *A tenuis*.

**Figure 3:**
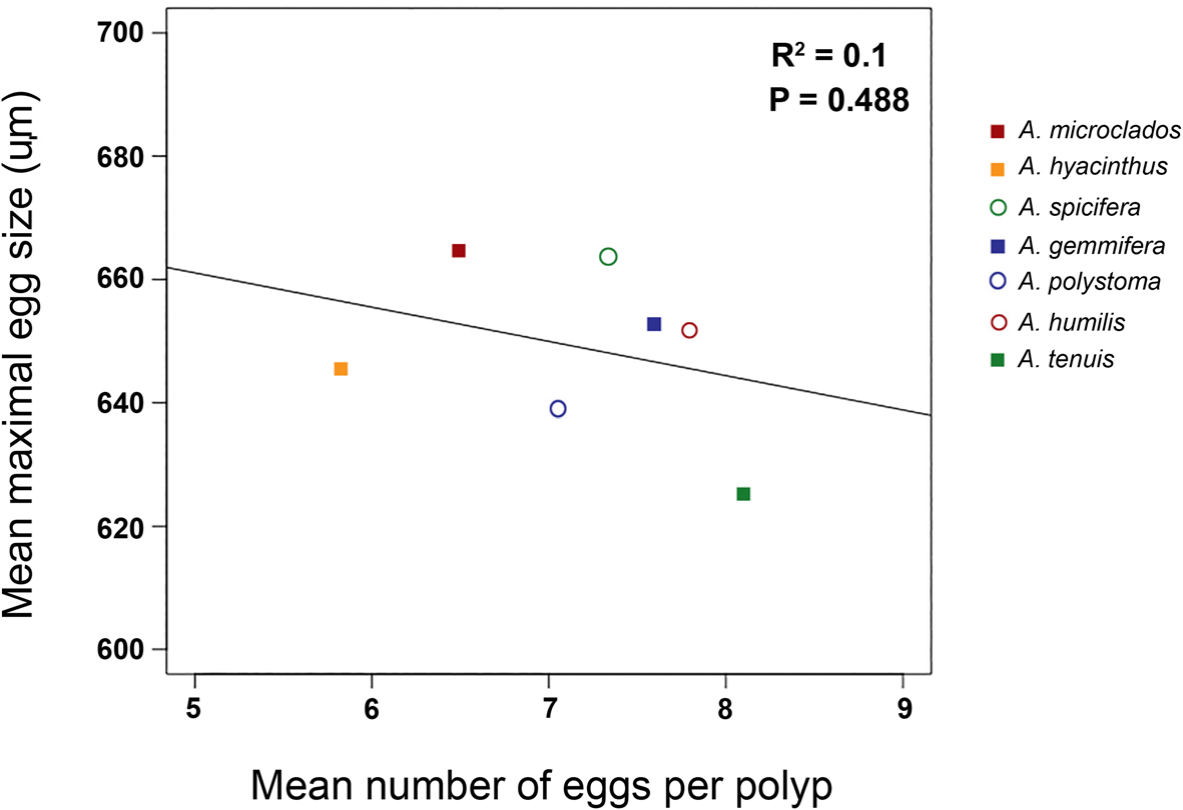
Regression analysis of mean egg size and mean number of eggs per polyp for seven *Acropora* species.

Egg sizes ranged from ~ 450 to 800 μm, with means of ~ 600 to 700 μm across all species and both seasons. Egg counts per polyp had high variability within each species, ranging from 4 to 12 eggs but with means ~ 6 to 8 eggs per polyp (Fig. 2). There was a poor correlation between polyp fecundity and mean egg sizes both within and between the seven *Acropora* species. In all but one species, egg size did not decrease significantly with increased fecundity (Fig. 2). In *A. spicifera*, mean egg sizes decreased significantly with increased fecundity (P = 0.002), but fecundity explained only ~ 8% of the variation in egg size. Within the other species, the relationship between fecundity and egg size was weak (R^2^ =  < 0.16) and insignificant (P > 0.05) (Fig. 2). Similarly, between the seven species there was no correlation between mean egg size and mean number of eggs (R^2^ = 0.10, P = 0.488) (Fig. 3).

### Seasonal effects on egg size and fecundity

**Figure 4:**
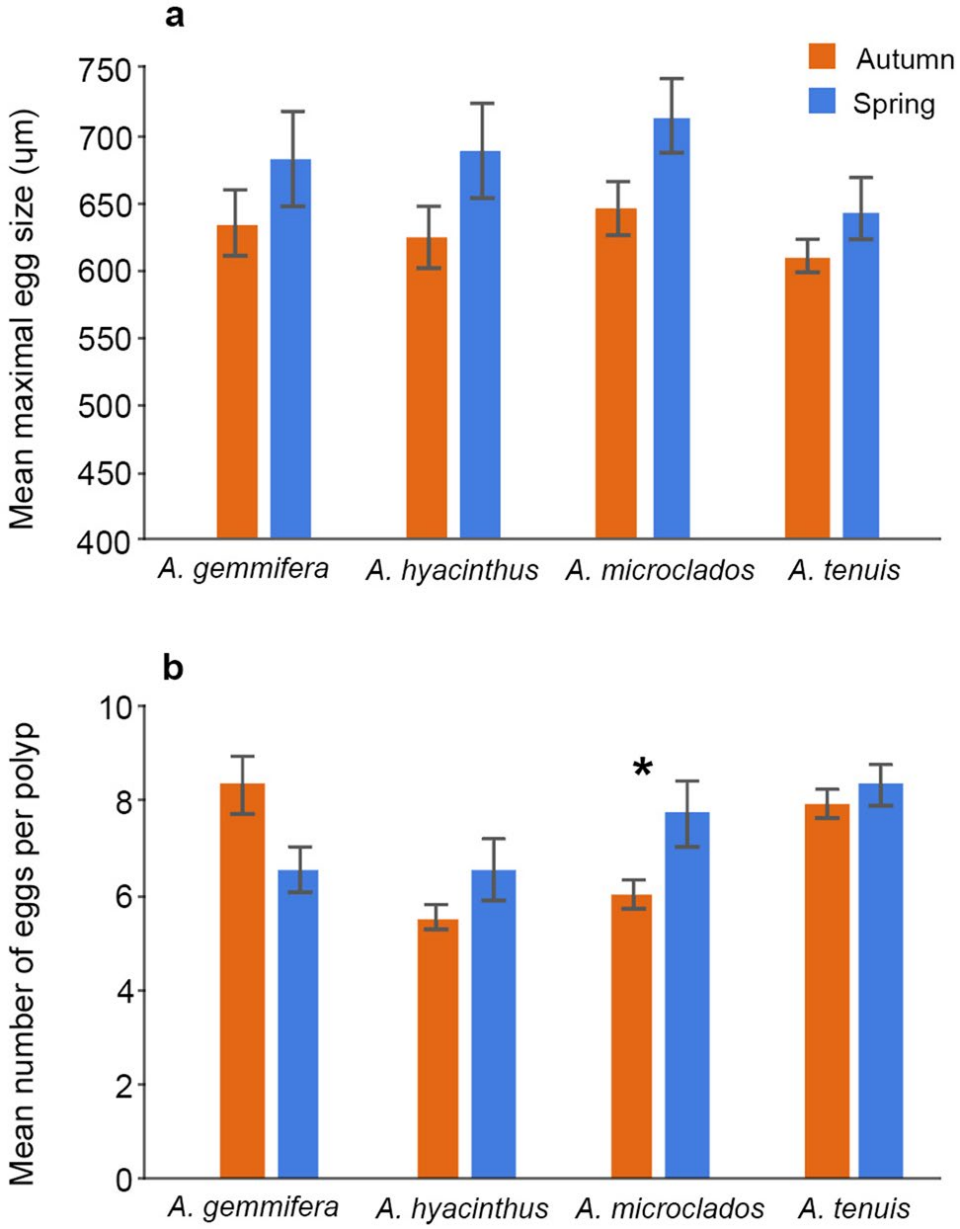
Mean (± SE) maximal egg size (**a**) and mean (± SE) number of eggs per polyp (**b**) in autumn compared with spring for the corals *Acropora gemmifera*, *A hyacinthus*, *A microclados* and *A tenuis*. Significant differences denoted by *.

Four biannually spawning *Acropora* species (*A. gemmifera, A. hyacinthus, A. microclados and A. tenuis*) were used to examine seasonal variability in reproductive output. No differences in mean maximal egg size were observed between the autumn and spring spawning events, across all of the species studied (Fig. 4, Supplementary Table S3). Similarly, there were no differences in the number of eggs per polyp in all species; the exception was *A. microclados*, which had higher fecundity in spring compared to autumn.

## Discussion

Many *Acropora* corals at Scott Reef spawn biannually, but most individuals spawn either in autumn or in spring (not in both seasons) and are thus temporally isolated from one another with respect to reproduction^10,25^. Gametogenesis takes ~ 4 to 6 months in *Acropora* corals at Scott Reef^10^. While it is unknown whether gametogenesis occurs at different rates in the different seasons, coral colonies experience different environmental conditions through the gametogenic period, leading up to spawning in the spring and autumn spawning seasons. Gametogenesis occurs through austral winter and early spring prior to the spring spawning (October/November), when water temperatures are cooler and days are shorter. Conversely, gametogenesis occurs through summer prior to the autumn spawning (March/April), when water temperatures are warmest (potentially stressfully warm), the days are longest, and tropical cyclones occur. Both spring and autumn spawning correspond with seasonal minimums in wind speed^26,27^. Within the thermal tolerance limits of the coral, warmer water temperatures and longer days theoretically increase energy availability for reproductive processes through increased metabolic activity and elevated photosynthesis^28,29^. Despite the different environmental conditions through gametogenesis, there were no seasonal differences in fecundity and eggs size observed in the biannually spawning *Acropora* corals studied here. There are several possibilities for the lack of seasonality observed in reproductive output. Firstly, fecundity and egg size varied widely within species, which confounded inferences about whether reproductive output was higher in a particular season. Other studies have similarly reported high variability, particularly in fecundity. Fecundity can vary widely with season^22,30^ and between years^31^, but there is also high variation between colonies, within a single colony^22^, with colony age^30^ and between colonies at different depths^13^. Fecundity can also vary in response to stressors^13^, however, there was no evidence of environmental stress, such as damaging waves from cyclones or heat stress causing coral bleaching^32^, before or during the period when samples were collected for this study. Adaptive plasticity in egg size (in response to conditions parents are exposed to), is discussed further below. Secondly, Scott Reef is situated in the tropics (14°S) with relatively small seasonal variations in temperature and day length, and has a light regime that is affected by high cloud cover during the summer cyclone season. Water temperatures are 2–4 °C cooler in the winter months leading up to spring spawning than in the summer months prior to autumn spawning. Day length is 1–2 h shorter in winter compared to summer, however summer cyclones and rainfall mean that overall sunshine hours are higher in the winter months. Consequently, there are cooler temperatures with more sunshine hours in the months leading to the spring spawning and warmer temperatures with less sunshine hours in the months leading to the autumn spawning, which may result in comparable available energy for reproduction during both spawning events. Thirdly, seasonal differences in environmental conditions may indeed drive some seasonal differences in energetics, but these could be channeled into other life history processes, such as calcification^12,33^, rather than fecundity and egg size. Variation in available energy may also affect egg quality rather than size or number. For example, in other invertebrates (greenlip abalone), while the size of the eggs do not increase, the density of protein and lipids increase throughout the spawning season^34^ and may indicate an increase in the quality over size of the egg. However, a study on the reef-building coral *Montipora capitata*, reported stable egg quality (lipids and antioxidants) regardless of the environmental conditions the parent colonies were exposed to, although egg sizes were not presented in this work^35^. The higher polyp fecundity in spring observed in *A. microclados* may have been an adaptive response to cooler (less favourable) conditions in spring. That is, an increase in parental investment to increase survival in less favourable conditions^36^. Alternatively, more sunshine hours during winter gametogenesis may have provided additional energy to produce more eggs in this species.

Early work on egg size and number of eggs suggests a simple trade-off model. That is, assuming resources for reproduction are limited, then an increase in gamete size should result in a reduction in the number of gametes^37^. Correspondingly, earlier studies of different coral species, genera and morphologies reported an inverse relationship between coral egg sizes and the number of eggs (fecundity)^21–23^, also suggesting that energy is channelled to either fewer large eggs or many small eggs^19,20^. However, in these cases, the reductions in fecundity with egg size among genera were attributed to the differences in polyp morphology (and sometimes reproductive mode i.e. brooder vs spawner). That is, differences in polyp size and structure can also affect egg size and fecundity^38,39^ independently of energetics. In our between species comparison, we did not see an inverse relationship between egg size and number of eggs (Fig. 3), but there were also no large differences in corallite size for the seven *Acropora* species studied here (see Supplementary Table S4 for corallite sizes of our species). However, it is important to note that the differences in reproductive mode and morphology (including polyp structure and size) between genera and species, interferes with the egg size versus number of eggs comparison in the context of a trade-off model. In order to determine if there is a trade-off between egg size and number of eggs, we need to look at individuals within a species. That is, do individuals with large eggs have fewer eggs than individuals with smaller eggs of the same species? We have been unable to locate any other dataset providing a within species comparison. Our study demonstrates that there is no direct relationship between egg size and fecundity, within these species of *Acropora*, and suggests that there is more than just a simple trade-off in resources influencing these measures.

Egg size has been shown to be a phenotypically plastic trait, regulated by the conditions the parent colony is exposed to. For example, a study on the broadcast spawning ascidian, *Styela plicata*, demonstrated that parents maintained at high densities produced smaller eggs, presumably reflecting the higher sperm concentrations expected at high adult densities, and therefore reduced requirement for a large target^40^. While the study was unable to measure the number of gametes, and provide an egg size versus number of eggs comparison, it did suggest that egg size is an adaptive plastic response, rather than a simple energetic constraint. Several studies have also reported varying effects of stress on the number and size of eggs. Under temperature stress sufficient to cause bleaching, corals within the same species can produce either fewer eggs (and maintain size) or smaller eggs (and maintain numbers) depending on their zooxanthellae clade and lipid levels^15^. Corals exposed to elevated nutrients levels also adjusted their reproductive output, with nitrogen reducing both egg size and number of eggs, and phosphorus producing smaller, but more eggs^41^. Furthermore, when coral colonies are transplanted to different latitudes, they adjust their egg size to be similar to local colonies. A transplant study conducted in Taiwan reported that coral colonies transplanted to higher latitudes and cooler waters, developed larger eggs, similar to local colonies, as an increased investment response to unfavourable conditions^36^. This phenotypic plasticity may allow for a type of maternal ‘bet-hedging’, where parents increase within clutch variation in offspring phenotype in response to unpredictable environmental conditions^42^. The results of our study showed high within species natural variability, but this variability was not consistent with the trade-off model. That is, while there may have been both large and small mature eggs within a species, the large eggs did not necessarily correspond with fewer eggs in a polyp. Within species size variation amongst offspring has traditionally been underestimated^43^, however, since offspring size can affect dispersal potential, producing a range of sizes, could spread offspring through a range of habitats, thereby spreading the risk of reproductive failure^44^.

It is often assumed that if resources are limited for reproduction, then an increase in egg size should result in a reduction in the number of eggs^37^. However, there are no datasets directly comparing egg size and number within coral species. We have shown that in seven *Acropora* coral species this trade-off between size and number did not occur. We also did not see any seasonal differences in these measures. We recorded high natural variability in both mature egg size and fecundity, a factor that should not be overlooked when using these measures to gauge or compare reproductive output (e.g. between seasons, years, locations). Since egg size and fecundity are affected by parent colony energy reserves, energy allocation to a range of other life history processes (e.g. growth and repair), polyp size and morphology, responses to environmental conditions, and the interaction of these factors, it is unlikely that there is a simple trade-off between size and number of eggs. It is also unlikely that these measures are constrained only by energetics, given the adaptive phenotypic plasticity reported in other studies^36, 40^. Furthermore, parental investment can come in the form of increased egg quality (e.g. lipids or antioxidants), rather than size or number of eggs. More research into coral energetics, natural variability, and adaptive plasticity is required to determine the mechanisms behind some of the patterns we observed, but our study doesn’t support a simple trade-off model in coral reproduction.

## Methods

### Study site and environmental conditions

Scott Reef is an isolated system of reefs located on the edge of the continental shelf approximately 270 km off the northwest Australian coastline (14°04′S, 121°46′E) (Supplementary Figure S1). Field samples were collected from six long-term monitoring locations across the reef system at depths ranging from 3 to 9 m^27^. Key environmental conditions at Scott Reef were summarized to examine seasonal differences in fecundity and egg size. Mean monthly values for environmental parameters were obtained for Scott Reef or Broome if parameters for Scott Reef were unavailable, and included: the mean monthly day lengths for Scott Reef (2016) from the US Naval Observatory (https://aa.usno.navy.mil/data/docs/Dur_OneYear.php), wind speed data for Scott Reef from the NOAA Earth Systems Laboratory (1968–1996) and water temperatures from the Hadley Centre Sea Surface Temperature dataset (1990–2000). All other parameters (daily sunshine hours, number of clear days, solar exposure, number of cloudy days and rainfall) were from the Australian Bureau of Meteorology Broome weather station (https://www.bom.gov.au/climate/averages/tables/cw_003003.shtml). The Broome weather station data were collected over the following years; daily sunshine hours from 1993 to 2016, number of clear days from 1939 to 2010, daily solar exposure from 1990 to 2017, number of cloudy days from 1939 to 2010 and rainfall from 1939 to 2017.

### Field samples

Colonies were sampled prior to the predicted mass spawning dates in autumn (March/April) and spring (October/November) in 2008, 2009, and 2010. Only sexually mature (> 20 cm diameter) colonies were used and three samples were collected from each colony. Samples were collected from the centre of the colony to avoid sterile colony margins. During fieldwork, colonies were examined in situ to rank the stages of egg development^27^ and confirm their participation in the coming spawning event. Eggs were scored according to the following observations; score 1: large pigmented (red or pink) eggs were clearly visible within the polyp, indicating that the colony would participate in the next spawning event within a month, score 2: large unpigmented (white or cream) eggs were clearly visible within the polyps, indicating that the colony would spawn within two months, score 3: small unpigmented (white or cream) eggs were visible within polyps, indicating the colony was unlikely to spawn for several months, and score 4: no eggs were visible within polyps, indicating that the colony had recently spawned or would not spawn for many months. Only mature egg samples were used in this study. Seven species of broadcast spawning *Acropora* corals (*A. gemmifera*, *A. humilis*, *A. hyacinthus*, *A. microclados*, *A. polystoma*, *A. spicifera* and *A. tenuis*) were sampled over three years (2008, 2009, and 2010) from up to six sites at Scott Reef (Supplementary Figure S2). All seven species were used to determine whether there were relationships between egg size and fecundity. Of these species, some spawn only in autumn (*A. humilis, A. polystoma* and *A. spicifera*) and others participate in both spawning seasons (*A. gemmifera*, *A. hyacinthus*, *A. microclados* and *A. tenuis*)^10^. The four species that spawn during both spawning seasons were used to analyze seasonal differences in egg size and fecundity. Between 15 and 112 (median = 21) colonies per species were sampled in total (Supplementary Table S1). For seasonal comparisons, replication ranged from 5 to 17 (median = 12) colonies per species, per season (Supplementary Table S2).

### Laboratory samples

Following collection in the field, *Acropora* samples were stored in a solution of 90% seawater and 10% formalin. Then in the lab, samples were decalcified in a solution of hydrochloric acid, formaldehyde (37%) and water. The initial solution was 5% HCl and 10% formaldehyde, but with a gradual increase in HCl from 5 to 10% over a period of 2–3 weeks. After decalcification, the tissue samples were stored in 70% ethanol. The tissue samples were then examined to determine the number and size of eggs in each polyp, using a Leica MS205 stereoscope. Five polyps were dissected for each of the three branches sampled per colony. The polyps were sampled from the centre sections of the branch to avoid the sterile growing tips. All of the eggs within each polyp were counted and the maximal diameter measured using Leica Application Suite version 3.1 software. If no eggs were present a further ten polyps were checked to confirm the results.

### Data analysis

Data collected in 2008, 2009 and 2010 were used to compare egg counts and egg sizes between the seasons (autumn and spring). Data were checked for normality using the Shapiro–Wilk test and for equality of variance using Levene’s test. Two sample *t* tests were used to compare the means of egg counts per polyp and maximal egg size for each of the species between autumn and spring spawning. Where data did not meet assumptions of normality, data were log transformed (*A. hyacinthus* egg count data). The correlations between mean maximal egg sizes and counts per polyp within and between species, were determined using regression analyses. To avoid the potentially confounding factor of season, only autumn samples were used for regression analyses in the four biannually spawning coral species. All tests and analyses were conducted in the software R.

## Supplementary information

Supplementary Information.

## Data Availability

Environmental data are available at https://aa.usno.navy.mil/data/docs/Dur_OneYear.php and https://www.bom.gov.au/climate/averages/tables/cw_003003.shtml. Egg size and fecundity data are available from authors upon reasonable request.
