# Supplementary material for: Egg size and fecundity of biannually spawning corals at Scott Reef
Source: Sci Rep. 2020 Jul 23;10:12313. doi: 10.1038/s41598-020-68289-4 (PMC7378250; doi:10.1038/s41598-020-68289-4)
Supplement: Supplementary file 1 — Supplementary Information. [file 41598_2020_68289_MOESM1_ESM.docx]

Egg size and fecundity of biannually spawning corals at Scott Reef

Taryn Foster^1*^ and James Gilmour^1^

^1^Australian Institute of Marine Science, Indian Ocean Marine Research Centre, University of Western Australia, Perth, Western Australia

* Corresponding author: t.foster@aims.gov.au

**Supplementary Information**

**
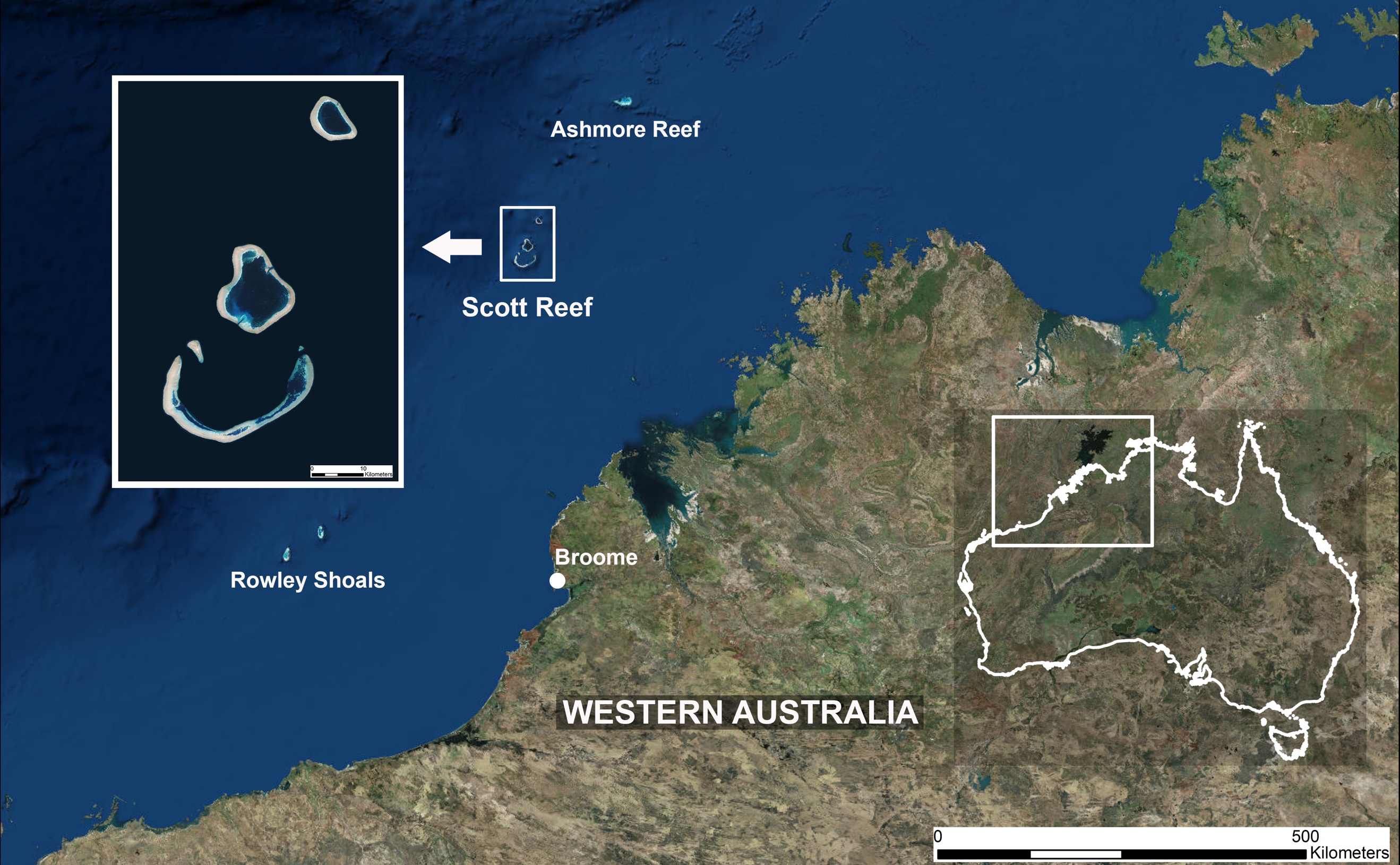
**

**Supplementary Figure S1.** Site map showing the location of the Scott Reef atoll system and other Kimberley Oceanic Reefs (Rowley Shoals and Ashmore Reef) in relation to north-western Australia. Map accessed in ESRI, ArcGIS.

**
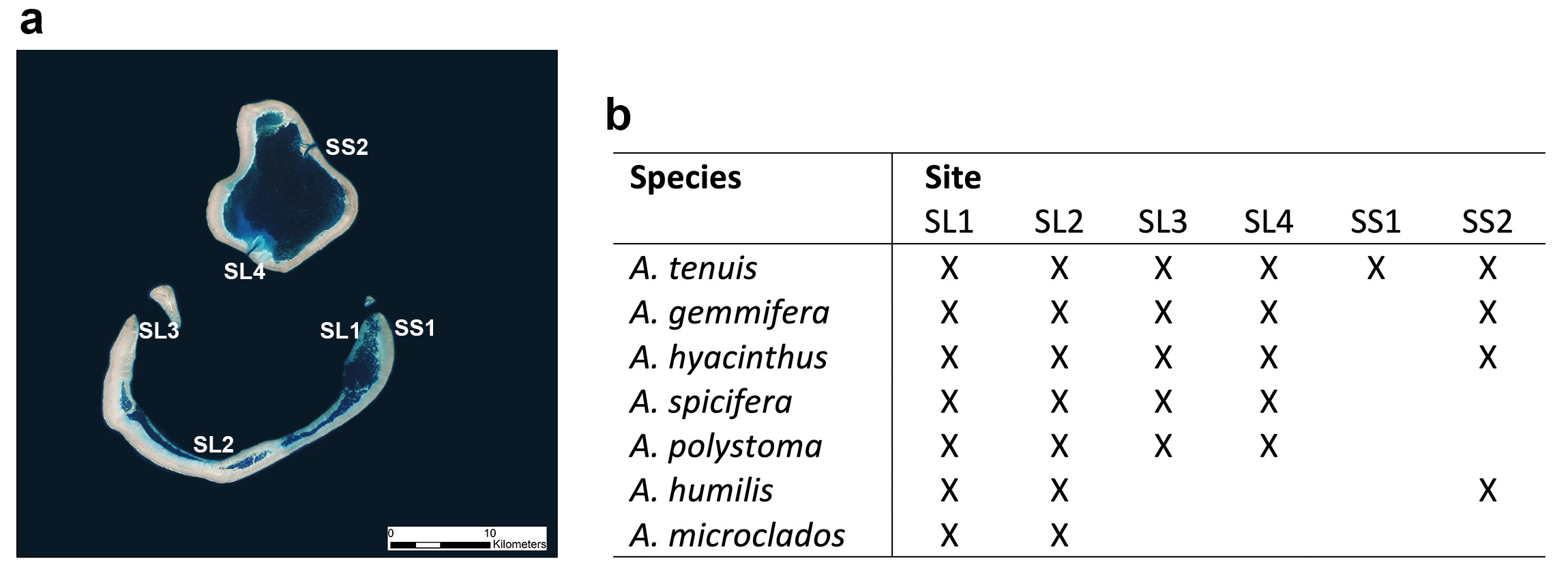
**

**Supplementary Figure S2.** Location of study sites at Scott Reef (**a**), and species sampled at each site (**b**). Map accessed in ESRI, ArcGIS.

**Supplementary Table S1.** The sample sizes (n), mean and standard error values for the egg counts and egg sizes for 7 *Acropora* coral species from Scott Reef.

| **Species** | **n** | **Egg Count** | **Egg Size** |
| --- | --- | --- | --- |
|  |  | **(mean ±** **SE)** | **(mean ±** **SE)** |
| *Acropora gemmifera* | 21 | 7.6 ± 0.46 | 652.8 ± 19.87 |
| *Acropora humilis* | 25 | 7.8 ± 0.26 | 651.7 ± 13.12 |
| *Acropora hyacinthus* | 20 | 5.8 ± 0.29 | 645.4 ± 19.61 |
| *Acropora microclados* | 17 | 6.5 ± 0.35 | 664.6 ± 17.81 |
| *Acropora polystoma* | 15 | 7.0 ± 0.36 | 639.2 ± 15.85 |
| *Acropora spicifera* | 112 | 7.3 ± 0.17 | 663.7 ± 7.31 |
| *Acropora tenuis* | 31 | 8.1 ± 0.26 | 625.3 ± 13.07 |

**Supplementary Table S2.** The sample sizes (n), mean and standard error values for the egg counts and egg sizes for 4 *Acropora* coral species that spawn in both autumn (March/April) and spring (October/November) from Scott Reef.

| **Species** | **Autumn** |  |  | **Spring** |  |  |
| --- | --- | --- | --- | --- | --- | --- |
|  | n | Egg Count  (mean ± SE) | Egg Size  (mean ± SE) | n | Egg Count  (mean ± SE) | Egg Size  (mean ± SE) |
| *Acropora gemmifera* | 13 | 8.3 ± 0.63 | 634.2 ± 23.29 | 8 | 6.5 ± 0.48 | 683.0 ± 35.24 |
| *Acropora hyacinthus* | 13 | 5.5 ± 0.26 | 623.7 ± 22.11 | 7 | 6.5 ± 0.62 | 688.9 ± 34.99 |
| *Acropora microclados* | 12 | 6.0 ± 0.32 | 644.2 ± 20.46 | 5 | 7.7 ± 0.67 | 713.5 ± 26.52 |
| *Acropora tenuis* | 17 | 7.9 ± 0.29 | 609.6 ± 13.30 | 14 | 8.3 ± 0.46 | 644.2 ± 23.62 |

**Supplementary Table S3.** Two sample t-test results comparing the egg count and egg size of spring (October/November) and autumn (March/April) spawning *Acropora* corals at Scott Reef. Significant (p < 0.05) seasonal differences are indicated with bold text.

| **Species** | **p** | **t** | **df** | |
| --- | --- | --- | --- | --- |
|  | | | | |
| **Egg Count** |  |  | |  |
| *Acropora gemmifera* | 0.066 | 1.952 | | 19 |
| *Acropora hyacinthus* | 0.079 | -1.861 | | 18 |
| *Acropora microclados* | **0.020** | -2.593 | | 15 |
| *Acropora tenuis* | 0.388 | -0.876 | | 29 |
|  |  |  | |  |
| **Egg Size** |  |  | |  |
| *Acropora gemmifera* | 0.243 | -1.204 | | 19 |
| *Acropora hyacinthus* | 0.110 | -1.676 | | 19 |
| *Acropora microclados* | 0.075 | -1.915 | | 15 |
| *Acropora tenuis* | 0.192 | -1.334 | | 29 |

**Supplementary Table S4**. Corallite size range for the study species. Sizes are from the Coral Traits Database (coraltraits.org).

| **Species** | **Corallite width** |
| --- | --- |
|  |  |
| *Acropora gemmifera* | 1 - 1.6 mm |
| *Acropora humilis* | 1 – 1.8 mm |
| *Acropora hyacinthus* | 0.4 – 1.1 mm |
| *Acropora microclados* | 0.6 – 1.2 mm |
| *Acropora polystoma* | 0.8 – 1.5 mm |
| *Acropora spicifera* | 0.5 - 1.1 mm |
| *Acropora tenuis* | 0.8 – 1.2 mm |
